# Atrioventricular block after acute myocardial infarction and its association with other clinical parameters in Pakistani patients: an institutional perspective

**DOI:** 10.1186/s13104-018-3431-5

**Published:** 2018-05-21

**Authors:** Kashif Ali Hashmi, Amir Shehzad, Atif Ali Hashmi, Amir Khan

**Affiliations:** 1 Chaudhry Pervaiz Elahi Institute of Cardiology Multan, Multan, Punjab Pakistan; 2 Liaquat National Hospital and Medical College, Karachi, Sindh Pakistan; 3 Kandahar University, Kandahar, Afghanistan

**Keywords:** Myocardial infarction, ST segment elevation myocardial infarction, Complete atrioventricular block

## Abstract

**Objectives:**

Conduction defects complicating acute myocardial infarction are frequently associated with increased morbidity and mortality. As frequency of this complication has not been widely studied in our population, therefore in this study we aimed to evaluate the frequency of complete atrioventricular block in patients with acute ST segment elevation myocardial infarction and its association with other clinical parameters.

**Results:**

The mean age of the patients was 50.55 ± 6.72 years at the time of MI. There were 147 (82.1%) males and 32 (17.9%) females. There were 83 (46.4%) patients having hypertension, 61 (34.1%) diabetes mellitus, 75 (41.9%) smokers, 75 (41.9%) patients having positive family history, 11 (6.1%) having dyslipidemia, and 73 (40.8%) obese patients in this study. The Frequency of complete atrioventricular (AV) block in acute ST segment elevation myocardial infarction was found to be 7.3%, and no association with any other clinical factor was found which could predict this condition according to results of our study. Therefore, protocols should be designed in our routine clinical practice to deal with such a life threatening condition.

## Introduction

Acute myocardial infarction (AMI) is a grave clinical condition and remains a leading cause of mortality all over the world. It occurs due to loss of blood supply to part of the heart muscle resulting in tissue injury as a result of oxygen deprivation [1]. Myocardial infarction is considered part of a spectrum referred to as acute coronary syndrome (ACS). The ACS continuum represents ongoing myocardial ischemia or injury and it consists of unstable angina, non-ST-segment elevation myocardial infarction (NSTEMI), and ST-segment elevation myocardial infarction (STEMI) [2]. Worldwide, more than 3 million people suffer from STEMIs and 4 million develop NSTEMIs a year [3]. STEMIs occur about twice as often in men as women [4].

The presence of conduction defects complicating acute myocardial infarction (MI) is relatively frequent and is associated with increased short and long term mortality rates [5].Atrioventricular (AV) block is a common complication of acute Myocardial Infarction (MI). In pre-thrombolytic era, high (second or third degree) AV block was seen in patients presenting with acute MI [6]. Although, the advent of thrombolytic therapy has substantially decreased the mortality associated with acute MI, the incidence of AV block persists [7]. Bhalli MA et al. [8] has found complete atrioventricular (AV) block in 8.1% patients presenting with acute myocardial infarction.

The rationale of this study is to evaluate the frequency of complete atrioventricular (AV) block in patients with acute ST segment elevation myocardial infarction (STEMI). As AV block along with acute myocardial infarction is associated with higher morbidity and mortality, so the results of this study will help the clinicians to design a protocol and make some clinical recommendations in our routine practice guidelines for management of these high risk group patients in order to reduce the morbidity and mortality of the community.

## Main text

It was a descriptive cross-sectional study conducted at department of cardiology Chaudhry Pervaiz Elahi Institute of Cardiology, Multan from January 2015 till July 2016 over a period of 1.5 years. All those patients diagnosed on admission as acute ST segment elevation Myocardial infarction (ST segment elevation of > 1 mm in limb leads and > 2 mm in precardial leads) were included in the study. Total number of 179 patients, fulfilling the inclusion criteria was selected after taking permission from ethical review committee and informed written consent to be included in the study from each patient. In all patients ECG was done which was evaluated by the consultant cardiologist (at least post fellowship experience of 5 years) for presence or absence of AV block in these particular patients. All this data was recorded on a predesigned proforma.

### Methods

Statistical analysis was performed using SPSS version 20.0. Mean and standard deviation were calculated for quantitative variables like age and duration of presentation. Frequency and percentage was calculated for qualitative variables like gender, obesity and complete AV block, smoker, hypertension, dyslipidemia, diabetes mellitus and family history. Effect modifiers like age, gender, duration of presentation, obesity, smoker, hypertension, dyslipidemia, family history of IHD and diabetes mellitus were controlled through stratifications. Post-stratification Chi square test was applied to see their effects on the outcome and P value ≤ 0.05 was considered as significant.

### Results

A total number of one hundred and seventy-nine (179) patients with ST segment elevation Myocardial Infarction were included in this study. The mean age of the patients was 50.55 ± 6.72 years at the time of MI. There were 147 (82.1%) males and 32 (17.9%) females in this study. The mean duration of presentation to hospital after myocardial infarction was 9.05 ± 5.14 h. There were 83 (46.4%) patients having hypertension, 61 (34.1%) diabetes mellitus, 75 (41.9%) smokers, 75 (41.9%) patients having positive family history, 11 (6.1%) having dyslipidemia, and 73 (40.8%) obese patients in this study. Complete atrioventricular block was found in 13 (7.3%) patients and shown in Table 1. No significant association was found between presence of AV block and various clinical parameters studied including age, gender, smoking, hypertension, diabetes, dyslipidemia, family history, obesity and duration of symptoms (Table 2).

**Table 1:** Demographic and clinical features of patients involved in the study

	N (%)
Age^a^	50.55 ± 6.72
Duration of presentation^a^	9.05 ± 5.14
Gender	
Male	147 (82.1)
Female	32 (17.9)
Hypertension	
Present	83 (46.4)
Absent	96 (53.6)
Diabetes	
Present	61 (34.1)
Absent	118 (65.9)
Smoking	
Present	75 (41.9)
Absent	104 (58.1)
Family history	
Present	41 (22.9)
Absent	138 (77.1)
Dyslipidemia	
Present	11 (6.1)
Absent	168 (93.9)
Obesity	
Present	73 (40.8)
Absent	106 (59.2)
Complete AV block	
Present	13 (7.3)
Absent	166 (92.7)

^a^ Mean ± SD

**Table 2:** Co-relation of atrioventricular block with other clinical features

	Complete AV block N		P value
	Present	Absent	
Age group (years)			
30–48	5	56	0.39*
49–54	6	55	
> 55	2	55	
Gender			
Male	10	137	0.61*
Female	3	29	
Smoking			
Present	5	70	0.794*
Absent	8	96	
Hypertension			
Present	5	78	0.55*
Absent	8	88	
Diabetes			
Present	3	58	0.38*
Absent	10	108	
Dyslipidemia			
Present	1	10	0.81*
Absent	12	156	
Family history			
Present	1	40	0.17*
Absent	12	126	
Obesity			
Present	4	69	0.44*
Absent	9	97	
Duration of presentation (h)			
1–6	2	62	0.22*
7–10	7	56	
11–24	4	48	

Chi square test was applied

P value ≤ 0.05 considered as significant

* Not significant at 0.05 level

Out of 179 patients, 38 (21.2%) patients presented within 12 h of onset of symptoms and therefore received thrombolytic therapy or angioplasty with stent placement. On the other hand remaining 141 (78.7%) patients presented to the hospital after 12 h of onset of symptoms and therefore received only conservative management including beta blockers, morphine and statins. All the 13 patients who developed AV block were those who received only conservative therapy. Among 13 patients who developed AV block, 9 patients (69.2%) had placement of permanent pacemakers; on the other hand, 4 patients (30.4%) received conservative management including antibiotic treatment or withdrawal of drugs. The duration of study follow up was only during the hospital stay, therefore long term outcome couldn’t be evaluated.

Complete atrioventricular (AV) block is a common complication of acute ST segment elevation myocardial infarction. There is an increased risk for the development of complete AV block in patients suffering from STEMI as compared to patient’s non-STEMI [9, 10]. The exact mechanism for the development of complete AV block still remains unclear. The 1st one may be cardio-inhibitory reflex originating from vagal reflexes in the ischemic left ventricular infero-posterior wall [11], the 2nd one is the AV nodal ischemia [12, 13].

Previous studies have shown that acute myocardial infarction complicated by complete AV block have a high killip class and increased risk of in hospital complications e.g. cardiogenic shock, intra-ventricular arrhythmias, and increased risk of in hospital mortality [14–16]. Experience with complete AV block in patients with STEMI have shown that CHB respond to atropine and isoproterenol in the vast majority of cases and usually it does not require even temporary pacing and almost never implantation of permanent pacemakers [17, 18]. However current guidelines have recommended that temporary pacing is indicated for symptomatic bradyarrythmias unresponsive to medical treatment [19].

The rate complete AV block after STEMI varies from 2.9 to 12.8%. In Pakistan the rate of complete AV block after STEMI have been reported to be 2.9–11.8% [20–22], this rate is less as compared to the other studies in the rate is reported to be 9.1–12.8% [23, 24]. In present study the rate of complete AV block was 7.3% in patients of STEMI. In our study, mean age of the patients was 50.55 ± 6.72 years at the time of MI but in the study of Iqbal et al. the main age of the patients was 58.88 ± 12.5 years that was higher as compared to the patients of our study [20].

We done stratification of various confounder variables e.g. age, gender, diabetes, hypertension, smoking, dyslipidemia and duration of presentation after MI to see is there any effect of these on the development of complete AV block after MI but we do not found any significant effect of these variables and the incidence of complete AV block remained same in all these groups.

## Limitations

The main limitation of the study was that this was a single institution study in which we found the frequency of complete atrioventricular (AV) block after acute ST segment elevation myocardial infarction to be 7.3%. However, as this is a major center of cardiovascular diseases in the province and represents patient population from both rural and urban areas, therefore we suggest that, protocols should be designed in our routine clinical practice to deal with such a life threatening condition.

## Abbreviations

AV block: complete atrioventricular block
MI: myocardial infarction
